# Characteristics and outcomes of acute pulmonary embolism among patients with polyvascular, single-vascular or no atherosclerotic disease: insights from RIETE

**DOI:** 10.1007/s00392-025-02706-4

**Published:** 2025-06-30

**Authors:** Silvia Cardi, Stefano Barco, Simon Wolf, Pablo Demelo-Rodríguez, Montserrat Pérez-Pinar, Andris Skride, Zoubida Tazi-Mezalek, Juan Bosco López-Sáez, Pablo Javier Marchena, Manuel Monreal, The RIETE investigators, P. Agudo, J. Aibar, A. Alberich-Conesa, A. Alda-Lozano, J. Alfonso, A. Alzueta-Álvarez, C. Amado, M. Angelina-García, J. I. Arcelus, A. Ballaz, R. Barba, C. Barbagelata, M. Barrón, B. Barrón-Andrés, C. S. Bonavila, R. Carrero, V. M. Castro-Bravo, G. Claver, C. De Juana-Izquierdo, J. Del Toro, P. Demelo-Rodríguez, A. M. Díaz-Brasero, M. C. Díaz-Pedroche, J. A. Díaz-Peromingo, R. Díaz-Simón, A. Díez-de León, Á. Dubois-Silva, D. Durán, J. C. Escribano, C. Fernández-Capitán, J. L. Fernández-Reyes, M. A. Fidalgo, I. Francisco, C. Gabara, F. Galeano-Valle, F. García-Bragado, C. García-González, A. García-Ortega, O. Gavín-Sebastián, A. Gil-Díaz, C. Gómez-Cepeda, C. Gómez-Cuervo, J. G. González-Martínez, A. González-Munera, I. Gorostidi-Álvarez, J. Gorostidi-Pérez, E. Grau, L. Guirado, J. Gutiérrez-Guisado, L. Hernández-Blasco, L. Jara-Palomares, D. Jiménez, I. Jou, M. D. Joya, S. Láinez-Justo, A. Latorre-Díez, J. L. Lobo, L. López-Jiménez, P. López-Miguel, J. J. López-Núñez, A. López-Ruiz, J. B. López-Sáez, A. Lorenzo, O. Madridano, A. Maestre, P. J. Marchena, M. Martín-del Pozo, F. Martín-Martos, L. Mas-Maresma, J. M. Maza, M. I. Mercado, M. Monreal, L. Monzón, J. A. Nieto, M. J. Núñez-Fernández, M. C. Olivares, L. Ordieres-Ortega, M. Ortiz, S. Otálora, R. Otero, N. Pacheco-Gómez, J. Pagán, P. Parra-Caballero, J. M. Pedrajas, C. Pérez-Ductor, M. Pérez-Pinar, M. L. Peris, M. L. Pesce, J. A. Porras, R. Puchades, G. Puche, A. Rivas, F. Rivera-Cívico, A. Rivera-Gallego, A. Rodríguez-Cobo, N. Ruiz-Giménez, G. Salgueiro, T. Sancho, V. Sendín, P. Sigüenza, S. Soler, S. Suárez-Fernández, B. Suárez-Rodríguez, C. Tolosa, M. I. Torres, J. Trujillo-Santos, F. Uresandi, R. Valle, J. F. Varona, E. Vázquez, G. Vidal, A. Villalobos, P. Villares, C. Ay, S. Nopp, I. Pabinger, Q. Van Thillo, P. Verhamme, T. Vanassche, A. T. Rocha, H. H. B. Yoo, C. A. Jiménez-Echandía, A. C. Montenegro, J. Roa, J. Hirmerova, R. Hirmerova, S. Acassat, L. Bertoletti, M. Brehon, A. Bura-Riviere, J. Catella, R. Chopard, O. Espitia, I. Mahé, F. Moustafa, L. Plaisance, G. Poenou, I. Quéré, E. Versini, S. Schellong, Y. Jenab, A. Khodayari, F. Rashidi, P. Sadeghipour, F. Tahmasbi, S. Yadangi, B. Brenner, G. Kenet, I. Tzoran, A. Abenante, G. Barillari, M. Basaglia, A. Bazza, F. Bilora, D. Bissacco, B. Brandolin, R. Casana, M. M. Ciammaichella, F. Dentali, P. Di Micco, M. Giorgi-Pierfranceschi, D. Lambertenghi-Deliliers, F. Negro, A. Poz, P. Prandoni, P. Simioni, C. Siniscalchi, B. Taflaj, A. Visonà, B. Zalunardo, H. Cesnieks, A. Skride, M. Zicāns, Z. Tazi-Mezalek, S. Fonseca, R. Marques, J. Meireles, M. Bosevski, S. Barco, S. Cardi, L. Mazzolai, S. Wolf, D. J. Angiolillo, J. A. Caprini, A. Khalil, L. Ortega-Paz, J. Tafur, I. Weinberg, H. M. Bui

**Affiliations:** 1 Italy Department of Biomedical Sciences, Humanitas University, Via Rita Levi Montalcini 4, 20072 Pieve Emanuele, Milan, Italy; 2 IRCCS Humanitas Research Hospital, Via Manzoni 56, 20089 Rozzano, Milan, Italy; 3 Department of Angiology, University Hospital Zurich, Zurich, Switzerland; 4 University of Zurich, Zurich, Switzerland; 5 Center for Thrombosis and Hemostasis, University Hospital of the Johannes Gutenberg University Mainz, Mainz, Germany; 6 Department of Internal Medicine, Hospital General Universitario Gregorio Marañón, Madrid, Spain; 7 Department of Internal Medicine, Hospital General Virgen de La Luz, Cuenca, Spain; 8 Department of Cardiology, Pauls Stradiņš Clinical University Hospital, Riga, Latvia; 9 Department of Internal Diseases, Riga Stradiņš University, Riga, Latvia; 10 Department of Internal Medicine, Hématologie Clinique, Centre Hospitalo-Universitaire Ibn Sina, Université Mohammed V de Rabat, Rabat, Morocco; 11 Department of Internal Medicine, Hospital Universitario Puerto Real, Puerto Real, Cádiz, Spain; 12 Medicine and Surgery Department, Universidad de Cádiz, Cádiz, Spain; 13 Department of Internal Medicine, Parc Sanitari Sant Joan de Déu, Hospital General, Sant Boi de Llobregat, Barcelona, Spain; 14 Chair for the Study of Thromboembolic Disease, Faculty of Health Sciences, UCAM - Universidad Católica San Antonio de Murcia, Murcia, Spain; 15 CIBER Enfermedades Respiratorias (CIBERES), Madrid, Spain

**Keywords:** Pulmonary embolism, Venous thromboembolism, Atherosclerosis, Cardiovascular disease, Platelet aggregation inhibitors, Registries retrospective studies

## Abstract

**Background:**

The role of atherosclerosis in pulmonary embolism (PE) prognosis remains uncertain. Our study assesses characteristics and outcomes of acute PE patients according to the presence and extent of atherosclerotic disease.

**Methods:**

Using data from the RIETE registry, acute PE patients were classified into three groups based on personal history: (1) polyvascular atherosclerosis, (2) single vascular atherosclerosis, and (3) no symptomatic atherosclerosis. Primary outcomes included recurrent PE and venous thromboembolism (VTE), arterial events, major bleeding, and all-cause death. Hazard ratios (HR) and Kaplan–Meier curves for clinical outcomes were estimated using Cox regression models.

**Results:**

Among 47,578 acute PE patients, 1,040 had polyvascular, 6,191 single-vascular, and 40,347 no atherosclerosis. During a median follow-up of 331 days, Adverse outcomes were more frequent in patients with atherosclerosis (vs. no atherosclerosis), rising with the number of affected vascular territories. Recurrent PE rates were 2.8, 1.6, and 1.2 per 100 patient-years in the polyvascular, single-vascular, and no atherosclerosis groups. Multivariable analysis showed a dose-dependent relationship between atherosclerosis and recurrent PE risk, with HRs of 3.2 (95% CI 1.7–5.9) and 1.6 (95% CI 1.1–2.3) for polyvascular and single-vascular disease (vs. no atherosclerosis). The risk of all-cause death followed a similar trend, with HRs of 1.3 (95% CI 1.1–1.6) and 1.2 (95% CI 1.1–1.4), respectively. Major bleeding appeared to be influenced by overall health status and antithrombotic therapy intensity.

**Conclusion:**

Atherosclerosis in acute PE patients may serve as a marker of disease severity and lead independently to adverse outcomes, highlighting the importance of cardiovascular risk stratification

**Graphical Abstract:**

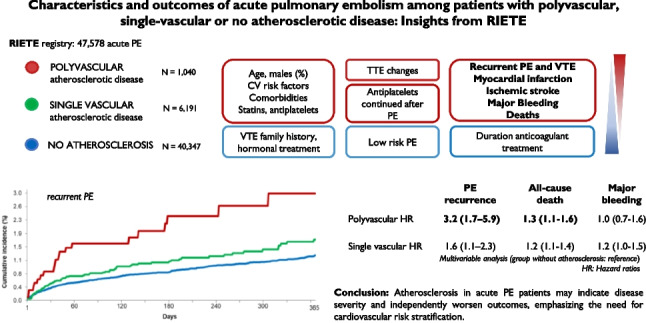

**Supplementary Information:**

The online version contains supplementary material available at 10.1007/s00392-025-02706-4.

## Introduction

Acute pulmonary embolism (PE) and atherosclerotic cardiovascular disease have traditionally been regarded as distinct conditions. Over the past decade, studies have shown that individuals with venous thromboembolism (VTE) exhibit a high prevalence of asymptomatic atherosclerosis and are characterized by a two- to three-times higher risk of developing arterial cardiovascular events compared to patients without VTE [1–7]. Moreover, patients with atherosclerosis may have a higher risk of suffering from VTE, particularly if the burden of atherosclerotic disease is larger [8–10].

The underlying mechanisms remain poorly understood. Conditions such as vasculitis, antiphospholipid syndrome, and patent foramen ovale are too rare to be the sole factors. Shared risk factors, such as older age, prothrombotic mutations, hormonal therapy, cancer, and classical cardiovascular risk factors, may predispose to both clinical manifestations [11–17]. The observation that statins and antiplatelet therapy reduce the risk of first-episode and recurrent VTE further supports a causal link [9, 18–23].

A recent retrospective analysis of German nationwide data found that patients with acute PE and symptomatic cardiovascular disease appear to have worse hospital outcomes compared to those without [24]. However, although a formal cardiovascular risk assessment is suggested in patients with PE, there is a lack of information on the characteristics and prognosis of patients with vs. without atherosclerotic disease, and whether the extent of cardiovascular burden would play a further prognostic role.

In this study from the Registro Informatizado Enfermedad TromboEmbolica (RIETE), we described the characteristics of clinical presentation, treatment, and clinical outcomes of acute PE patients with polyvascular, single vascular, or no atherosclerotic disease.

## Methods

### Data source

We did a patient-level analysis of the RIETE registry, an ongoing multinational multicentre registry that includes consecutive patients with acute VTE confirmed with imaging and a minimum of 3 months follow-up (NCT02832245) [25]. Data extracted from the registry undergo regular on-site monitoring, achieving an overall agreement of 95% between the registered information and patient records. In accordance with local Ethics Committee requirements, patients provided either written or oral consent to participate in the registry [25]. A statistical analysis plan was prepared before formal data analysis for this study. The Ethics Committee on Research from the Hospital Universitari Germans Trias i Pujol (Badalona, Spain) approved the study protocol on April 7, 2017 (code IRB00002131).

Patients diagnosed with PE between February 2009 (the date when all arterial variables were included into the database) and March 2024 were recruited, irrespective of the presence or absence of concomitant deep vein thrombosis (DVT). We stratified them into three groups according to prior clinical manifestations of atherosclerosis. These included: (1) patients with polyvascular atherosclerotic disease, defined as patients with a known personal history of symptomatic atherosclerosis, encompassing two or more of the following diseases: myocardial infarction or angina, cerebrovascular disease (ischemic stroke or transient ischemic attack, regardless of etiology), or peripheral artery disease, (2) patients with single vascular atherosclerotic disease; (3) patients without a prior symptomatic atherosclerotic disease [26].

The primary objective was to provide a comprehensive description of the demographic characteristics, distribution of comorbidities, cardiovascular and thromboembolic risk factors, clinical and radiological features of PE presentation, and treatments across these three groups. Furthermore, we studied the risk of experiencing clinical outcomes, including recurrent VTE, major bleeding, arterial cardiovascular events, and disease-specific death. Major bleeding events were defined according to the ISTH criteria [27]. Anticoagulation management followed the clinical practices of each participating institution, with details on the type, dose, and duration of treatment systematically recorded. Most outcomes were classified and reported from each participant sites.

### Statistical analysis

We used descriptive statistics to summarize baseline characteristics, prevalence of risk factors, comorbidities, and concomitant therapies. Data are presented either as count and percentages or in the case of continuous variables as mean and standard deviation (SD) or median and interquartile range (IQR). Additionally, we calculated the incidence rate of adverse events during follow-up as events per 100 patient-years. Cox proportional hazard regression models were used for univariable and multivariable analyses to estimate hazard ratios (HR) and the corresponding 95% confidence intervals (95% CI). Patients without history of atherosclerosis served as the reference group for comparative analyses. Two multivariable Cox regression models were used. Model 1 included age, sex, and anticoagulation duration. Model 2 additionally adjusted for renal failure, prior VTE, anemia, active cancer, chronic lung and heart disease, low oxygen saturation (≤ 90%), tachycardia (heart rate > 110 bpm), diabetes, and use of antiplatelet agents and statins. Kaplan–Meier survival curves were plotted to visualize and compare time-to-event distributions across groups (VTE and PE recurrence, major bleeding, and all-cause death). Statistical analyses were conducted using SPSS.

### Role of the funding source

The authors are solely responsible for the content of this work. No external funding was obtained for this study. The study statistician had full access to all the data. The corresponding author had the responsibility for submission for publication.

## Results

We included a total of 47,578 patients with acute PE: of these, 1,040 had polyvascular atherosclerotic disease, 6,191 had a single vascular atherosclerotic disease, and 40,347 had no history of atherosclerotic disease.

Among patients with polyvascular disease, prior myocardial infarction or angina and prior cerebrovascular disease exceeded 70%, whereas peripheral artery disease was described in 64%. Among patients with isolated atherosclerosis, prior myocardial infarction or angina and prior cerebral ischemia exceeded 40%, whereas peripheral artery disease was described in 17%: Table 1.

**Table 1:** Baseline characteristics of the study population

	Polyvascular atherosclerotic disease	Single vascular atherosclerotic disease	No history of atherosclerotic disease
*Patients, N*	1,040	6,191	40,347
Demographics			
Male sex, n/N (%)	672 (65)^‡^	3,320 (54)^‡^	19,085 (47)
Age (years), median (IQR)	79 (71–84)^‡^	77 (68–83)^‡^	68 (53–78)
BMI (kg/m2), median (IQR)	27 (25–30)^‡^	27 (25–31)	28 (25–31)
CV risk factors			
Current smoker, n/N (%)	141 (14)	706 (12)^‡^	5,482 (14)
Diabetes, n/N (%)	405 (39)^‡^	1,681 (27)^‡^	5,569 (14)
Arterial hypertension, n/N (%)	861 (83)^‡^	4,531 (74)^‡^	18,199 (45)
VTE risk factors			
History of VTE, n/N (%)	155 (15)	944 (15)^‡^	5,465 (14)
Family history of VTE, n/N (%)	10 (2.5)^‡^	97 (3.8)^‡^	1,365 (7.0)
Active cancer, n/N (%)	142 (14)^*^	985 (16)	6,717 (17)
Recent surgery, n/N (%)	99 (9.5)	596 (9.6)	4,211 (10)
Recent immobility ≥ 4 days, n/N (%)	340 (33)^‡^	1,727 (28)^‡^	8,068 (20)
Hormonal treatment, n/N (%)	19 (1.9)^‡^	108 (1.8)^‡^	2,763 (7.0)
Pregnancy, n/N (%)	0	4 (0.06)^‡^	184 (0.5)
Postpartum, n/N (%)	1 (0.1)	0	225 (0.6)
Arterial disease			
Prior MI or angina, n/N (%)	803 (77)	2,591 (42)	0
Prior ischemic stroke or TIA, n/N (%)	736 (71)	2,548 (41)	0
Peripheral artery disease, n/N (%)	668 (64)	1,052 (17)	0
Comorbidities			
Atrial fibrillation, n/N (%)	138 (20)^‡^	526 (12)^‡^	1,440 (4.9)
Chronic heart failure, n/N (%)	369 (36)^‡^	1,222 (20)^‡^	2,313 (5.7)
Chronic lung disease, n/N (%)	299 (29)^‡^	1,223 (20)^‡^	4,965 (12)
SAHS, n/N (%)	52 (5.0)^†^	277 (4.5)^‡^	1,273 (3.2)
Chronic renal failure, n/N (%)	440 (42)^‡^	2,131 (34)^‡^	7,534 (19)
Nephrotic syndrome, n/N (%)	10 (1)^†^	33 (0.5)	151 (0.4)
Periodic hemodialysis, n/N (%)	4 (0.4)^*^	13 (0.2)^*^	36 (0.1)
Liver cirrhosis, n/N (%)	9 (0.9)^*^	30 (0.5)	160 (0.4)
Liver steatosis, n/N (%)	23 (2.2)^†^	91 (1.5)^†^	431 (1.1)
Chronic liver disease (no biopsy), n/N (%)	8 (0.8)	71 (1.1)	383 (1.0)
Antiphospholipid syndrome, n/N (%)	1 (0.1)	5 (0.1)	55 (0.1)
Concomitant therapies			
Corticosteroids, n/N (%)	135 (14)^‡^	703 (12)^‡^	3,672 (9.8)
Anticoagulants, n/N (%)	72 (16)^‡^	318 (11)^‡^	1,119 (5.6)
NSAIDs, n/N (%)	82 (8.7)^*^	483 (8.4)^‡^	2,527 (6.8)
Antiplatelets, n/N (%)	724 (74)^‡^	3,671 (62)^‡^	3,956 (11)
Erythropoietin, n/N (%)	10 (1.2)^*^	47 (1.0)^†^	185 (0.5)
Statins, n/N (%)	639 (63)^‡^	3,039 (50)^‡^	7,598 (19)

Legend: BMI: body mass index; CrCl: creatinine clearance (CrCl < 50 mL/min); CV: cardiovascular; MI: myocardial infarction; NSAIDs: non-steroidal anti-inflammatory drugs; SAHS: sleep apnoea hypopnea syndrome; VTE: venous thromboembolism

Comparisons between subgroups of patients: ^*^p < 0.05, ^†^*p* < 0.01; ^‡^*p* < 0.001

### Baseline characteristics

Patients with polyvascular atherosclerotic disease were older: median age was 79 (IQR: 71–84) years vs. 77 (IQR: 68–83) years among patients with single vascular atherosclerotic disease vs. 68 (IQR: 53–78) years among patients without prior symptomatic atherosclerosis. Patients with atherosclerotic disease had a higher prevalence of classical cardiovascular risk factors, such as male sex, diabetes, arterial hypertension, chronic lung disease, renal failure, atrial fibrillation, particularly if two or more territories were affected. Statins, antiplatelet agents, corticosteroids, anticoagulants (before index PE), and erythropoietin were also progressively more prevalent. The prevalence of smoking and the median body mass index at baseline appeared similar across groups; Table 1.

The prevalence of classical risk factors for VTE appeared similar across the three groups with respect to prior VTE, cancer, and recent surgery. In contrast, prolonged immobility, hormonal treatment, and family history of VTE were less prevalent with an increasing number of territories affected by atherosclerotic disease.

### Presentation of acute PE and treatment

The proportion of patients with initial oxygen saturation below 90% was higher in the group of patients with polyvascular atherosclerotic disease (34% vs. 29% with single vascular atherosclerotic disease, vs. 23% with no history of atherosclerotic disease). A similar distribution was found for the proportion of patients with hypotension (4.1% vs. 3.9% vs. 3.0%). Consistently, patients with polyvascular and single vascular disease were less frequently classified as low-risk according to ESC criteria for pulmonary embolism (14% and 19%, respectively), compared to those without atherosclerotic disease (33%); Table 2.

**Table 2:** Clinical and radiological presentation of pulmonary embolism

	Total	Polyvascular atherosclerotic disease	Single vascular atherosclerotic disease	No history of atherosclerotic disease
*Patients*, N		1,040	6,191	40,347
Clinical presentation				
HR, mean ± SD		88 ± 21^‡^	89 ± 21^‡^	92 ± 20
RR, mean ± SD		22 ± 7.0^‡^	21 ± 6.6^‡^	20 ± 6.4
SBP, mean ± SD		129 ± 26	130 ± 25	129 ± 23
SBP < 90 mmHg, n/N (%)		43 (4.1)^*^	244 (3.9)^‡^	1,216 (3.0)
SatO_2_, mean ± SD		91 ± 6.4^‡^	91 ± 7.2^‡^	92 ± 6.5
SatO_2_ < 90%, n/N (%)	26,717	210 (34)^‡^	1,037 (29)^‡^	5,165 (23)
PE ESC risk class				
Low risk	46,673	143 (14)^‡^	1,140 (19)^‡^	13,141 (33)
Intermediate-low risk	46,673	769 (74)^‡^	4,342 (71)^‡^	22,453 (57)
Intermediate-high risk	46,673	78 (7.6)	409 (6.7)	2,695 (6.8)
High risk	46,673	43 (4.2)	244 (4.0)^‡^	1,216 (3.1)
Laboratory tests				
Total cholesterol, mean ± SD	18,238	158 ± 41^‡^	161 ± 43^‡^	177 ± 47
HDL, mean ± SD	12,553	42 ± 34	42 ± 67^*^	44 ± 31
LDL, mean ± SD,	11,645	93 ± 34^‡^	96 ± 38^‡^	110 ± 37
Triglycerides, mean ± SD	16,577	129 ± 61	132 ± 70	134 ± 71
Compression ultrasonography		**584**	**3,466**	**23,693**
Positive (DVT), n/N (%)		353 (60)	1,918 (55)^‡^	14,345 (61)
Proximal, n/N (%)		267 (76)	1,493 (78)	11,096 (77)
Distal, n/N (%)		66 (19)	337 (18)	2,482 (17)
Helical CT scan		**869**	**5,349**	**36,172**
Segmental and subsegmental, n/N (%)		241 (27.8)	1447 (27.1)	9566 (26)
Lobar, n/N (%)		224 (26)	1,324 (25)	9,201 (25)
Main, n/N (%)		183 (21)^*^	1,240 (23)	8,722 (24)
Central, n/N (%)		50 (5.8)^*^	372 (7.0)^†^	2,969 (8.2)
RV/LV ratio, mean ± SD	3,633	1.1 ± 0.37	1.1 ± 0.31^*^	1.1 ± 0.36
Echocardiogram		**530**	**3,111**	**20,502**
PAP mean ± SD		47 ± 15^†^	46 ± 17^‡^	44 ± 16
Persistent PFO, n/N (%)		8 (3.8)^†^	43 (3.7)^‡^	102 (1.2)
Right atrium dilatation, n/N (%)		163 (36)^‡^	707 (27)^‡^	3,795 (23)
Right ventricular hypokinesis, n/N (%)		116 (26)^*^	589 (23)^*^	3,568 (21)
Right ventricular hypertrophy, n/N (%)		39 (16)^‡^	147 (10)	902 (8.9)
RVDD/LVDD ratio ≥ 1.0, n/N (%)	3,611	33 (39)	156 (35)	1,085 (35)
TAPSE (mm), mean ± SD	11,187	19 ± 4.8^‡^	19 ± 5.1^‡^	20 ± 5.2

Legend: DVT: deep vein thrombosis; HDL: high density lipoprotein; HR: heart rate (beats/min); LDL: low density lipoprotein; LVDD: left ventricular diameter; PAP: pulmonary artery pressure (mmHg); PFO: patent foramen ovale; RR: respiratory rate (breaths/min); RV/LV ratio: right ventricle to left ventricle ratio; RVDD: right ventricular diameter; SatO2: oxygen saturation (%); SBP: systolic blood pressure (mmHg);; TAPSE: Tricuspid annular plane systolic excursion (mm)

Comparisons between subgroups of patients: ^*^p < 0.05, ^†^*p* < 0.01; ^‡^*p* < 0.001

Approximately half of the patients was screened for the presence of concomitant DVT, the distribution of which was similar across groups. Echocardiography was also performed in approximately half of the patients: key findings are summarized in Table 2, indicating a higher prevalence of patent foramen ovale, right atrium dilatation, and right ventricular hypertrophy in patients with atherosclerotic disease.

Cholesterol, LDL-C, and triglycerides levels were lower in patients with polyvascular vs. single vascular vs. no atherosclerotic disease, reflecting the prevalent use of lipid-lowering therapies.

Table 3 summarized the characteristics of initial and long-term treatment. Patients with atherosclerotic disease were more often treated with antiplatelet therapies after acute PE (23% vs. 21% vs. 2.5%, respectively), but the median length of anticoagulation was slightly lower (155 vs. 182 vs. 190 days, respectively).

**Table 3:** Treatment

	Polyvascular atherosclerotic disease	Single vascular atherosclerotic disease	No history of atherosclerotic disease
*Patients, N*	1,040	6,191	40,347
Initial treatment			
LMWH, n/N (%)	849 (82)	5,200 (84)^†^	33,344 (83%)
UFH, n/N (%)	78 (7.5)	452 (7.3)^*^	2,626 (6.5%)
DOACs, n/N (%)	62 (6.0)	236 (3.8)^‡^	2,219 (5.5%)
Thrombolytic, n/N (%)	8 (0.8)^‡^	126 (2.0)^†^	1,077 (2.7%)
Fondaparinux, n/N (%)	20 (1.9)	96 (1.6)	723 (1.8%)
No anticoagulant drugs, n/N (%)	4 (0.4)	20 (0.3)	92 (0.2%)
Vasopressors, n/N (%)	10 (1.0)	62 (1.0)	328 (0.8%)
ECMO, n/N (%)	1 (0.1)	6 (0.1)	51 (0.1%)
Surgical (or catheter), n/N (%)	12 (1.2)	73 (1.2)	545 (1.4%)
Long term treatment			
Median duration of anticoagulation days, median (Q1-Q3)	155 (93–329)^‡^	182 (96–367)^‡^	190 (103–374)
DOACs, n/N (%)	200 (19)^‡^	1,241 (20)^‡^	9,743 (24)
VKAs, n/N (%)	491 (47)	2,849 (46)	18,447 (46)
LMWH, n/N (%)	278 (27)	1,682 (27)	10,520 (26)
Other drugs, n/N (%)	11 (1.1)	81 (1.3)	428 (1.1)
No anticoagulant drugs, n/N (%)	13 (1.3)^†^	49 (0.8)^‡^	168 (0.4)
Surgical (or catheter), n/N (%)	2 (0.2)	13 (0.2)	66 (0.2)
Antiplatelets continued after VTE, n/N (%)	225 (23)^‡^	1,245 (21)^‡^	933 (2.5)

Legend: DOACs: direct oral anticoagulants; ECMO: extracorporeal membrane oxygenation LMWH: low-molecular-weight heparin; UFH: unfractionated heparin.

Comparisons between subgroups of patients: ^*^p < 0.05, ^†^*p* < 0.01; ^‡^*p* < 0.001

### Clinical outcomes

Patients with polyvascular atherosclerotic disease experienced the highest annual rates of recurrent VTE, with progressively lower rates observed in those with single-territory or no atherosclerotic disease; Table 4. Such trend was driven by recurrent PE events: 2.8, 1.6, and 1.2 per 100 patient-years among patients with polyvascular, single vascular, and without atherosclerotic disease, respectively. Similar trends were documented for myocardial infarction (2.4 vs. 0.8 vs. 0.2 per 100 patient-years), ischemic stroke (1.7 vs. 1.1 vs. 0.4 per 100 patient-years), and major bleeding (5.6 vs. 5.0 vs. 3.2 per 100 patient-years). The leading causes of bleeding were gastrointestinal and intracranial.

**Table 4:** Outcomes during anticoagulation

	Polyvascular atherosclerotic disease		Single vascular atherosclerotic disease		No history of atherosclerotic disease	
	*N*	*Events per 100* *patient-years (IC 95%)*	*N*	*Events per 100 patient-years (IC 95%)*	*N*	*Events per 100* *patient-years (IC 95%)*
*Patients, N*	1,037		6,178		40,291	
Recurrent PE	23	2.8 (1.8–4.1)^‡^	88	1.6 (1.3–1.9)^*^	460	1.2 (1.1–1.3)
Recurrent VTE	31	3.8 (2.6–5.3)^†^	132	2.4 (2.0–2.8)	809	2.1 (2.0–2.3)
Major bleeding	47	5.6 (4.2–7.4)^‡^	281	5.0 (4.5–5.6)^‡^	1,214	3.2 (3.0–3.4)
Gastrointestinal	20	2.4 (1.5–3.6)^‡^	86	1.5 (1.2–1.9)^‡^	362	0.94 (0.8–1.0)
Intracranial	12	1.4 (0.8–2.4)^*^	66	1.2 (0.9–1.5)^‡^	243	0.6 (0.6–0.7)
Retroperitoneal	3	0.4 (0.1–1.0)	22	0.4 (0.3–0.6)^*^	83	0.2 (0.2–0.3)
Vaginal	0	0.0 (0.0–0.4)	3	0.1 (0.0–0.1)	53	0.1 (0.1–0.2)
Other hematoma	5	0.6 (0.2–1.3)	54	1.0 (0.7–1.2)	274	0.7 (0.6–0.8)
Myocardial infarction	20	2.4 (1.5–3.6)^‡^	46	0.8 (0.6–1.1)^‡^	75	0.2 (0.2–0.2)
Ischemic stroke	14	1.7 (1.0–2.7)^‡^	60	1.1 (0.8–1.4)^‡^	161	0.4 (0.4–0.5)
Limb amputation	1	0.1 (0.0–0.6)	12	0.2 (0.1–0.4)^‡^	8	0.0 (0.0–0.0)
Death	207	24.6 (21.4–28.1)^‡^	983	17.4 (16.3–18.5)^‡^	3,797	9.8 (9.5–10.1)
Fatal PE	22	2.6 (1.7–3.9)^‡^	91	1.6 (1.3–2.00)^‡^	270	0.7 (0.6–0.8)
Fatal bleeding	13	1.5 (0.9–2.6)^‡^	52	0.9 (0.7–1.2)^‡^	165	0.4 (0.4–0.5)
Fatal MI	8	1.0 (0.4–1.8)^‡^	10	0.2 (0.1–0.3)^‡^	14	0.0 (0.0–0.1)
Fatal ischemic stroke	5	0.6 (0.2–1.3)^‡^	11	0.2 (0.1–0.3)^†^	26	0.1 (0.0–0.1)
Disseminated cancer	31	3.7 (2.6–5.2)	224	4.0 (3.5–4.5)	1,534	4.0. (3.8–4.2)
Heart failure	23	2.7 (1.8–4.0)^‡^	65	1.2 (0.9–1.5)^‡^	148	0.4 (0.3–0.5)

Legend: DVT: deep vein thrombosis; PE: pulmonary embolism; VTE: venous thromboembolism.

Comparisons between subgroups of patients: ^*^p < 0.05, ^†^*p* < 0.01; ^‡^*p* < 0.001

Patients with polyvascular disease had the highest rate of death at one year (24.6 per 100 patient-years) followed by patients with single vascular atherosclerotic disease (17.4 per 100 patient-years) and no atherosclerotic disease (9.8 per 100 patient-years). Fatal cardiovascular events, encompassing PE, myocardial infarction, and stroke, and bleeding events contributed to this trend. The cancer-specific death rate was similar across groups.

### Univariable and multivariable time-to-event Cox regression models

Having a polyvascular or single vascular atherosclerotic disease was associated with recurrent PE and with all-cause death. This association remained significant after adjustment for age, sex, and length of anticoagulation (Model 1) and after full adjustment for several comorbidities and antiplatelet and statin use (Model 2). By increasing the number of conditioning variables, the strength of association between atherosclerotic diseases progressively increased for the outcome recurrent PE: HR 3.2 (95%CI 1.7–5.9) for polyvascular disease vs. no atherosclerotic disease and HR 1.6 (95%CI 1.1–2.3) for single vascular atherosclerotic disease vs. no atherosclerotic disease in Model 2. In contrast, it progressively reduced for the outcome death: HR 1.3 (95%CI 1.1–1.6) for polyvascular disease vs. no atherosclerotic disease and HR 1.2 (95%CI 1.1–1.4) for single vascular atherosclerotic disease vs. no atherosclerotic disease in Model 2; Table 5.

**Table 5:** Time-to-event Cox regression analysis

	Polyvascular disease	Single vascular artery disease	Absence of prior atherosclerotic events
Recurrent VTE			
Univariate model	1.7 (1.2–2.4)	1.1 (0.9–1.3)	Reference
Adjusted model 1	1.8 (1.3–2.7)	1.2 (1.0–1.5)	Reference
Adjusted model 2	2.2 (1.3–3.7)	1.3 (1.0–1.8)	Reference
Recurrent PE			
Univariate model	2.2 (1.4–3.3)	1.3 (1.0–1.6)	Reference
Adjusted model 1	2.4 (1.5–3.6)	1.4 (1.1–1.8)	Reference
Adjusted model 2	3.2 (1.7–5.9)	1.6 (1.1–2.3)	Reference
Major Bleeding			
Univariate model	1.6 (1.2–2.2)	1.6 (1.4–1.8)	Reference
Adjusted model 1	1.2 (0.9–1.6)	1.3 (1.1–1.4)	Reference
Adjusted model 2	1.0 (0.7–1.6)	1.2 (1.0–1.5)	Reference
All-cause death			
Univariate model	2.3 (2.0–2.7)	1.8 (1.6–1.9)	Reference
Adjusted model 1	1.4 (1.2–1.6)	1.2 (1.1–1.3)	Reference
Adjusted model 2	1.3 (1.1–1.6)	1.2 (1.1–1.4)	Reference

Legend PE: pulmonary embolism; VTE: venous thromboembolism

Adjusted model 1 (age, sex, length of anticoagulation)

Adjusted model 2 (age, sex, length of anticoagulation, renal failure (CrCl < 50 mL/min), prior VTE, anemia, cancer, chronic lung disease, chronic heart failure, Sat ≤ 90%, heart rate > 110 bpm, diabetes, antiplatelets, statins)

**Fig. 1:**
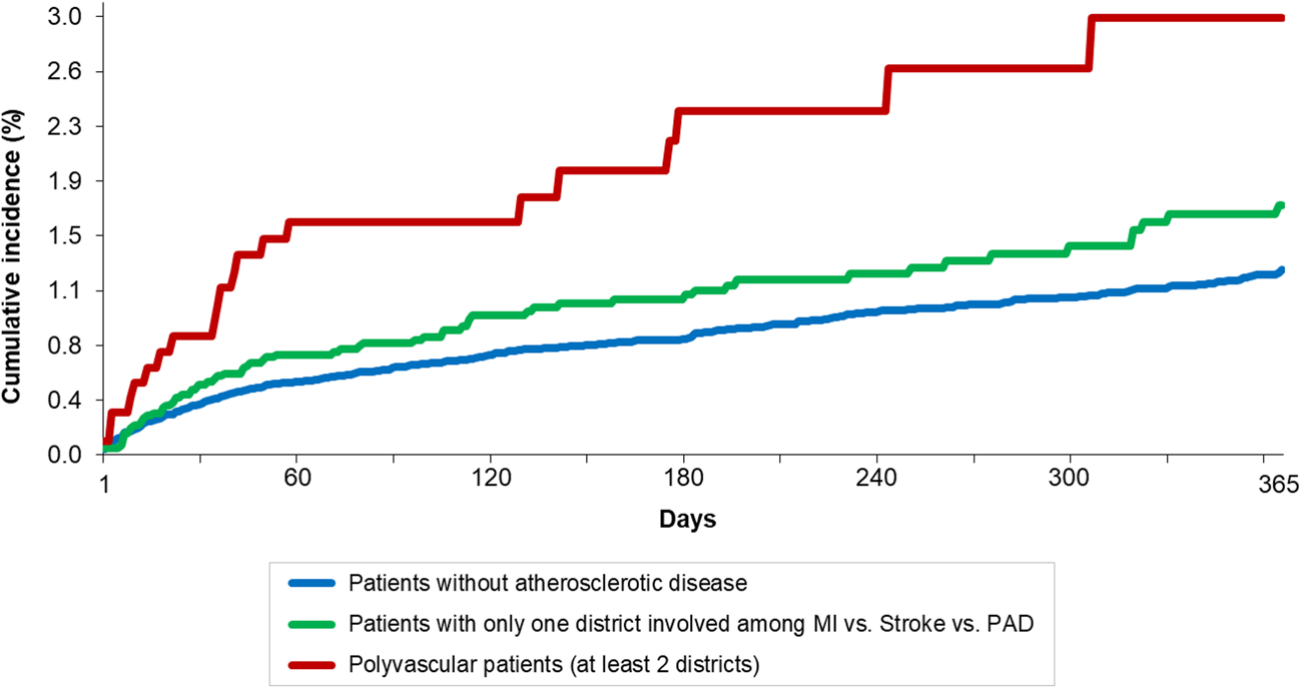
Kaplan–Meier estimates for pulmonary embolism (PE) recurrences over one year. This graph depicts the cumulative incidence of PE recurrence in patients without atherosclerotic disease (blue line), with single vascular artery disease (green line), and with polyvascular disease (red line). A log-rank test revealed statistically significant differences between the groups (*p*-value < 0.001)

Similarly to all cause death, the strength of association between polyvascular or single-vascular atherosclerosis and major bleeding progressively reduced by increasing the number of adjustment factors, particularly in patients with polyvascular disease: HR for major bleeding 1.6 (95%CI 1.2–2.2) at univariate analysis, HR 1.2 (95%CI 0.9–1.60) in Model 1, and HR 1.0 (95%CI 0.7–1.6) in Model 2. The Kaplan–Meier curve estimators illustrate the cumulative incidence of clinical events for each outcome; Table 5, Fig. 1 and Supplementary Figs. 1, 2 and 3.

## Discussion

In patients with acute PE and a history of atherosclerotic disease from the RIETE registry, compared to those without atherosclerosis, we observed a higher incidence of adverse outcomes, an increased risk of recurrent PE and, albeit modestly, all-cause death, with both findings exhibiting a dose-dependent relationship with the number of arterial beds affected by atherosclerosis: Graphical abstract. Major bleeding risk was not influenced by the presence or extent of atherosclerosis, but was instead associated with the patient's overall health status and the intensity of antithrombotic therapy. These results suggest that atherosclerotic disease, particularly as its burden increases, may serve as a prognostic marker for worse outcomes in patients with acute PE or, even, be implicated in the genesis of future adverse events.

When stratifying PE patients based on the presence and extent of atherosclerosis, significant baseline differences emerged in terms of age, risk factors, comorbidities, and treatment. Patients with polyvascular atherosclerosis, and to a lesser extent those with single-vascular involvement, tended to be older, predominantly male, and had a higher burden of comorbidities, contributing to poorer health status at the time of PE diagnosis. Whether these, or atherosclerosis and its burden itself, could increase the risk of adverse outcomes in these patients was the core issue.

The higher incidence of myocardial infarction and stroke observed in patients with atherosclerosis, particularly in those with multiple arterial beds involved, is consistent with existing literature, where polyvascular disease is recognized as an independent risk factor for major cardiovascular events [26, 28–32].

Advanced age, combined with the high prevalence of heart failure, atrial fibrillation, and other cardiovascular and VTE risk factors in these patients, likely contributed to the heightened thrombotic risk and may have driven the increased rates of VTE and recurrent PE in these groups [14, 33]. Similarly, older age and the high prevalence of comorbidities that significantly affect prognosis, such as chronic kidney, lung, and hepatic diseases, along with heart failure, likely contributed to the higher rates of major bleeding and mortality observed in patients with atherosclerosis. Furthermore, over 20% of these patients were receiving concomitant antiplatelet and anticoagulation therapy, further elevating the risk of bleeding.

Cancer, which was equally prevalent across all groups, emerged as the leading cause of death, while PE was the primary cause of death in patients with single-vascular or no atherosclerosis and heart failure was the main cause in those with polyvascular disease.

To assess the independent contribution of atherosclerosis and its burden to adverse outcomes, we examined its association with PE recurrences, accounting for potential confounders. As additional variables were considered, the strength of this association increased, reinforcing that atherosclerosis is linked to an elevated risk of PE and VTE recurrences, particularly in patients with polyvascular disease.

This finding aligns with other studies, which have demonstrated that symptomatic atherosclerosis is associated with an increased risk of VTE [8–10, 34]. Notably, sub-analyses of the TRA2P-TIMI 50 and PEGASUS-TIMI 54 trials, which assessed different antiplatelet regimens in stable symptomatic atherosclerosis, found that the degree of atherosclerotic burden, particularly in polyvascular disease, correlated with a higher VTE risk [9]. These results are relevant, as polyvascular disease appears to increase the risk of both arterial and venous thrombosis compared to isolated atherosclerosis. In terms of recurrent VTE risk, both statins and antiplatelet agents have been shown to reduce primary and secondary VTE risks, indicating a potential role for atherosclerosis in recurrence, even in the absence of direct evidence [9, 18–20, 22, 23]. Our findings support this hypothesis, revealing a dose-dependent relationship between the number of affected arterial beds and the risk of recurrent PE and VTE. While the underlying mechanisms remain unclear, chronic systemic inflammation, endothelial dysfunction, and a prothrombotic state, common to both conditions, may contribute to this overlap [35–37].

In contrast to the strong association between atherosclerosis and recurrent VTE, the increased bleeding risk observed in the univariate analysis was not significant after full adjustment. This indicates that bleeding risk in this population is more likely related to advanced age, comorbidities, and antithrombotic therapy intensity, rather than the atherosclerotic disease burden itself.

Regarding all-cause death, while the association with atherosclerosis weakened after adjustment for confounders, it remained significant, demonstrating a modest but dose-dependent relationship, especially in patients with polyvascular disease, emphasizing its influence on outcomes in PE patients. This relationship between atherosclerosis and PE recurrence, as well as all-cause death, was further supported by our Kaplan–Meier curves, which demonstrated an increasing rate of VTE and PE recurrences and all-cause death with greater atherosclerotic burden.

Our findings underscore the importance of comprehensive cardiovascular risk assessment in all patients with acute PE, aligning with recent recommendations for cardiovascular evaluation at the three-month follow-up after PE diagnosis [38]. Also, for patients with known atherosclerotic disease, it is essential to ensure proper control of existing cardiovascular risk factors, adjusting treatment to meet guideline-recommended targets. Achieving LDL-cholesterol goals is particularly significant, as lipid-lowering therapies have been shown to reduce the risk of both primary and recurrent VTE [9, 18–23]. Additionally, PE patients with a history of symptomatic atherosclerotic disease, particularly those with polyvascular involvement, may benefit from extended anticoagulant therapy due to their increased risk of PE and VTE recurrence. However, to minimize the risk of bleeding, concomitant anticoagulant and antiplatelet therapies should be reserved for cases with clear indications and discontinued as soon as clinically warranted. This highlights the need for an individualized treatment approach.

The findings of this study should be interpreted in the light of its limitations. The RIETE registry relies on data entered by a variety of practitioners across multiple centres, which could introduce variability in the accuracy of the data. The definition of polyvascular disease was based on symptomatic events in major arterial territories, which likely underestimates the true burden of atherosclerosis by excluding asymptomatic disease and other vascular beds not routinely captured in the registry. In addition, the observational design of this study carries an inherent risk of selection bias, as patients with more severe atherosclerosis may have been more likely to be enrolled and subjected to more intensive monitoring. Although male sex was more prevalent in patients with polyvascular disease, the interaction between sex and atherosclerosis extent was not formally analyzed, as this was beyond the primary objectives of the study. However, sex was included in all multivariable models to mitigate confounding. Despite these limitations, the large, multinational nature of the RIETE registry provides a comprehensive and globally relevant perspective on the interaction between atherosclerosis and PE.

In conclusion, we showed that atherosclerotic disease worsens the prognosis in patients with acute PE, including the risk of recurrent PE and death. These risks were higher in patients with larger burden of atherosclerosis and only partially depended on age, sex, and comorbidities. This finding suggests that especially polyvascular arterial disease in patients with acute PE may serve as a marker of disease severity and also lead independently to adverse events. As per current consensus documents, a formal cardiovascular risk stratification is recommended in patients with acute PE.

## Supplementary Information

Below is the link to the electronic supplementary material.

Supplementary file1 (DOCX 103 KB)

## Data Availability

The data that support the findings of our study can be accessed through the RIETE registry upon request.

## References

[1] Agnelli G, Becattini C (2006) Venous thromboembolism and atherosclerosis: common denominators or different diseases? J Thromb Haemost 4(9):1886–1890. 10.1111/j.1538-7836.2006.02138.x 16961596 10.1111/j.1538-7836.2006.02138.x

[2] Prandoni P, Bilora F, Marchiori A, Bernardi E, Petrobelli F, Lensing AW et al (2003) An association between atherosclerosis and venous thrombosis. N Engl J Med 348(15):1435–1441. 10.1056/NEJMoa022157 12686699 10.1056/NEJMoa022157

[3] Hong C, Zhu F, Du D, Pilgram TK, Sicard GA, Bae KT (2005) Coronary artery calcification and risk factors for atherosclerosis in patients with venous thromboembolism. Atherosclerosis 183(1):169–174. 10.1016/j.atherosclerosis.2005.03.047 15939424 10.1016/j.atherosclerosis.2005.03.047

[4] Prandoni P, Ghirarduzzi A, Prins MH, Pengo V, Davidson BL, Sorensen H et al (2006) Venous thromboembolism and the risk of subsequent symptomatic atherosclerosis. J Thromb Haemost 4(9):1891–1896. 10.1111/j.1538-7836.2006.02058.x 16961597 10.1111/j.1538-7836.2006.02058.x

[5] Sorensen HT, Horvath-Puho E, Pedersen L, Baron JA, Prandoni P (2007) Venous thromboembolism and subsequent hospitalisation due to acute arterial cardiovascular events: a 20-year cohort study. Lancet 370(9601):1773–1779. 10.1016/S0140-6736(07)61745-0 18037081 10.1016/S0140-6736(07)61745-0

[6] Klok FA, Mos IC, Broek L, Tamsma JT, Rosendaal FR, de Roos A, Huisman MV (2009) Risk of arterial cardiovascular events in patients after pulmonary embolism. Blood 114(8):1484–1488. 10.1182/blood-2009-05-220491 19549987 10.1182/blood-2009-05-220491

[7] Keller K, Kohring C, Farmakis IT, Valerio L, Barco S, Batzing J et al (2023) Impact of venous thromboembolism on incidence of arterial thromboembolism - An analysis of German outpatient claims data. Thromb Res 226:9–17. 10.1016/j.thromres.2023.04.008 37079980 10.1016/j.thromres.2023.04.008

[8] Sorensen HT, Horvath-Puho E, Sogaard KK, Christensen S, Johnsen SP, Thomsen RW et al (2009) Arterial cardiovascular events, statins, low-dose aspirin and subsequent risk of venous thromboembolism: a population-based case-control study. J Thromb Haemost 7(4):521–528. 10.1111/j.1538-7836.2009.03279.x 19192118 10.1111/j.1538-7836.2009.03279.x

[9] Cavallari I, Morrow DA, Creager MA, Olin J, Bhatt DL, Steg PG et al (2018) Frequency, predictors, and impact of combined antiplatelet therapy on venous thromboembolism in patients with symptomatic atherosclerosis. Circulation 137(7):684–692. 10.1161/CIRCULATIONAHA.117.031062 29084737 10.1161/CIRCULATIONAHA.117.031062

[10] Piazza G, Goldhaber SZ, Lessard DM, Goldberg RJ, Emery C, Spencer FA (2011) Venous thromboembolism in patients with symptomatic atherosclerosis. Thromb Haemost 106(6):1095–1102. 10.1160/TH11-07-0469 22012325 10.1160/TH11-07-0469

[11] Ye Z, Liu EH, Higgins JP, Keavney BD, Lowe GD, Collins R, Danesh J (2006) Seven haemostatic gene polymorphisms in coronary disease: meta-analysis of 66,155 cases and 91,307 controls. Lancet 367(9511):651–658. 10.1016/S0140-6736(06)68263-9 16503463 10.1016/S0140-6736(06)68263-9

[12] Rosendaal FR, Van Hylckama VA, Tanis BC, Helmerhorst FM (2003) Estrogens, progestogens and thrombosis. J Thromb Haemost 1(7):1371–1380. 10.1046/j.1538-7836.2003.00264.x 12871270 10.1046/j.1538-7836.2003.00264.x

[13] Grilz E, Posch F, Konigsbrugge O, Schwarzinger I, Lang IM, Marosi C et al (2018) Association of Platelet-to-Lymphocyte Ratio and Neutrophil-to-Lymphocyte Ratio with the Risk of Thromboembolism and Mortality in Patients with Cancer. Thromb Haemost 118(11):1875–1884. 10.1055/s-0038-1673401 30296815 10.1055/s-0038-1673401

[14] Ageno W, Becattini C, Brighton T, Selby R, Kamphuisen PW (2008) Cardiovascular risk factors and venous thromboembolism: a meta-analysis. Circulation 117(1):93–102. 10.1161/CIRCULATIONAHA.107.709204 18086925 10.1161/CIRCULATIONAHA.107.709204

[15] Ageno W, Di Minno MN, Ay C, Jang MJ, Hansen JB, Steffen LM et al (2014) Association between the metabolic syndrome, its individual components, and unprovoked venous thromboembolism: results of a patient-level meta-analysis. Arterioscler Thromb Vasc Biol 34(11):2478–2485. 10.1161/ATVBAHA.114.304085 25212233 10.1161/ATVBAHA.114.304085 PMC4322778

[16] Mahmoodi BK, Cushman M, Anne Naess I, Allison MA, Bos WJ, Braekkan SK et al (2017) Association of Traditional Cardiovascular Risk Factors With Venous Thromboembolism: An Individual Participant Data Meta-Analysis of Prospective Studies. Circulation 135(1):7–16. 10.1161/CIRCULATIONAHA.116.024507 27831499 10.1161/CIRCULATIONAHA.116.024507 PMC5201424

[17] Delluc A, Lacut K, Rodger MA (2020) Arterial and venous thrombosis: What’s the link? A narrative review Thromb Res 191:97–102. 10.1016/j.thromres.2020.04.035 32416310 10.1016/j.thromres.2020.04.035

[18] Becattini C, Agnelli G, Schenone A, Eichinger S, Bucherini E, Silingardi M et al (2012) Aspirin for preventing the recurrence of venous thromboembolism. N Engl J Med 366(21):1959–1967. 10.1056/NEJMoa1114238 22621626 10.1056/NEJMoa1114238

[19] Agarwal V, Phung OJ, Tongbram V, Bhardwaj A, Coleman CI (2010) Statin use and the prevention of venous thromboembolism: a meta-analysis. Int J Clin Pract 64(10):1375–1383. 10.1111/j.1742-1241.2010.02439.x 20716146 10.1111/j.1742-1241.2010.02439.x

[20] Brighton TA, Eikelboom JW, Mann K, Mister R, Gallus A, Ockelford P et al (2012) Low-dose aspirin for preventing recurrent venous thromboembolism. N Engl J Med 367(21):1979–1987. 10.1056/NEJMoa1210384 23121403 10.1056/NEJMoa1210384

[21] Delluc A, Ghanima W, Kovacs MJ, Shivakumar S, Kahn SR, Sandset PM et al (2022) Statins for venous event reduction in patients with venous thromboembolism: A multicenter randomized controlled pilot trial assessing feasibility. J Thromb Haemost 20(1):126–132. 10.1111/jth.15537 34564938 10.1111/jth.15537

[22] Kunutsor SK, Seidu S, Khunti K (2017) Statins and secondary prevention of venous thromboembolism: pooled analysis of published observational cohort studies. Eur Heart J 38(20):1608–1612. 10.1093/eurheartj/ehx107 28369602 10.1093/eurheartj/ehx107 PMC5837543

[23] Farmakis IT, Christodoulou KC, Hobohm L, Konstantinides SV, Valerio L (2024) Lipid lowering for prevention of venous thromboembolism: a network meta-analysis. Eur Heart J. 10.1093/eurheartj/ehae361 38874212 10.1093/eurheartj/ehae361

[24] Keller K, Hobohm L, Munzel T, Ostad MA (2019) Impact of symptomatic atherosclerosis in patients with pulmonary embolism. Int J Cardiol 278:225–231. 10.1016/j.ijcard.2018.12.019 30558990 10.1016/j.ijcard.2018.12.019

[25] Bikdeli B, Jimenez D, Hawkins M, Ortiz S, Prandoni P, Brenner B et al (2018) Rationale, Design and Methodology of the Computerized Registry of Patients with Venous Thromboembolism (RIETE). Thromb Haemost 118(1):214–224. 10.1160/TH17-07-0511 29304541 10.1160/TH17-07-0511 PMC5821113

[26] Mazzolai L, Teixido-Tura G, Lanzi S, Boc V, Bossone E, Brodmann M, et al. (2024) ESC Guidelines for the management of peripheral arterial and aortic diseases. Eur Heart J. 2024. 10.1093/eurheartj/ehae179. 10.1093/eurheartj/ehae179 39210722

[27] Schulman S, Kearon C, (2005) Subcommittee on Control of Anticoagulation of the S, Standardization Committee of the International Society on T, Haemostasis. Definition of major bleeding in clinical investigations of antihemostatic medicinal products in non-surgical patients. J Thromb Haemost. 3(4):692–4. 10.1111/j.1538-7836.2005.01204.x 10.1111/j.1538-7836.2005.01204.x 15842354

[28] Alberts MJ, Bhatt DL, Mas JL, Ohman EM, Hirsch AT, Rother J et al (2009) Three-year follow-up and event rates in the international REduction of atherothrombosis for continued health registry. Eur Heart J 30(19):2318–2326. 10.1093/eurheartj/ehp355 19720633 10.1093/eurheartj/ehp355 PMC2755116

[29] van den Berg MJ, Bhatt DL, Kappelle LJ, de Borst GJ, Cramer MJ, van der Graaf Y et al (2017) Identification of vascular patients at very high risk for recurrent cardiovascular events: validation of the current ACC/AHA very high risk criteria. Eur Heart J 38(43):3211–3218. 10.1093/eurheartj/ehx102 28369481 10.1093/eurheartj/ehx102

[30] Subherwal S, Bhatt DL, Li S, Wang TY, Thomas L, Alexander KP et al (2012) Polyvascular disease and long-term cardiovascular outcomes in older patients with non-ST-segment-elevation myocardial infarction. Circ Cardiovasc Qual Outcomes 5(4):541–549. 10.1161/CIRCOUTCOMES.111.964379 22715460 10.1161/CIRCOUTCOMES.111.964379 PMC3707283

[31] van der Meer MG, Cramer MJ, van der Graaf Y, Appelman Y, Doevendans PA, Nathoe HM, Group SS (2014) The impact of polyvascular disease on long-term outcome in percutaneous coronary intervention patients. Eur J Clin Invest 44(3):231–239. 10.1111/eci.12222 24372467 10.1111/eci.12222

[32] Weissler EH, Jones WS, Desormais I, Debus S, Mazzolai L, Espinola-Klein C et al (2020) Polyvascular disease: A narrative review of current evidence and a consideration of the role of antithrombotic therapy. Atherosclerosis 315:10–17. 10.1016/j.atherosclerosis.2020.11.001 33190107 10.1016/j.atherosclerosis.2020.11.001

[33] Konstantinides SV, Meyer G, Becattini C, Bueno H, Geersing GJ, Harjola VP et al (2020) 2019 ESC Guidelines for the diagnosis and management of acute pulmonary embolism developed in collaboration with the European Respiratory Society (ERS). Eur Heart J 41(4):543–603. 10.1093/eurheartj/ehz405 31504429 10.1093/eurheartj/ehz405

[34] Christodoulou KC, Barco S, Abele C, Lodigiani C, Monreal M, Konstantinids SV, Valerio L. Burden of atherosclerosis and outcomes of acute pulmonary embolism: a post-hoc analysis of the Hokusai-VTE trial. J Am Heart Assoc. In press 10.1161/JAHA.125.041420 PMC12887200 41195787

[35] Migliacci R, Becattini C, Pesavento R, Davi G, Vedovati MC, Guglielmini G et al (2007) Endothelial dysfunction in patients with spontaneous venous thromboembolism. Haematologica 92(6):812–818. 10.3324/haematol.10872 17550854 10.3324/haematol.10872

[36] Simes J, Robledo KP, White HD, Espinoza D, Stewart RA, Sullivan DR et al (2018) D-Dimer predicts long-term cause-specific mortality, cardiovascular events, and cancer in patients with stable coronary heart disease: LIPID study. Circulation 138(7):712–723. 10.1161/CIRCULATIONAHA.117.029901 29367425 10.1161/CIRCULATIONAHA.117.029901

[37] Mahmoodi BK, Gansevoort RT, Veeger NJ, Matthews AG, Navis G, Hillege HL et al (2009) Microalbuminuria and risk of venous thromboembolism. JAMA 301(17):1790–1797. 10.1001/jama.2009.565 19417196 10.1001/jama.2009.565

[38] Klok FA, Ageno W, Ay C, Back M, Barco S, Bertoletti L et al (2022) Optimal follow-up after acute pulmonary embolism: a position paper of the European Society of Cardiology Working Group on Pulmonary Circulation and Right Ventricular Function, in collaboration with the European Society of Cardiology Working Group on Atherosclerosis and Vascular Biology, endorsed by the European Respiratory Society. Eur Heart J 43(3):183–189. 10.1093/eurheartj/ehab816 34875048 10.1093/eurheartj/ehab816 PMC8790766

