# Supplementary material for: Characteristics and outcomes of acute pulmonary embolism among patients with polyvascular, single-vascular or no atherosclerotic disease: insights from RIETE
Source: Clin Res Cardiol. 2025 Jun 30;115(3):472–83. doi: 10.1007/s00392-025-02706-4 (PMC12894142; doi:10.1007/s00392-025-02706-4)
Supplement: Supplementary file 1 — Supplementary file1 (DOCX 103 KB) [file 392_2025_2706_MOESM1_ESM.docx]

**Supplementary Figure 1. Kaplan Meier curves for Venous Thromboembolism (VTE) recurrence**

**
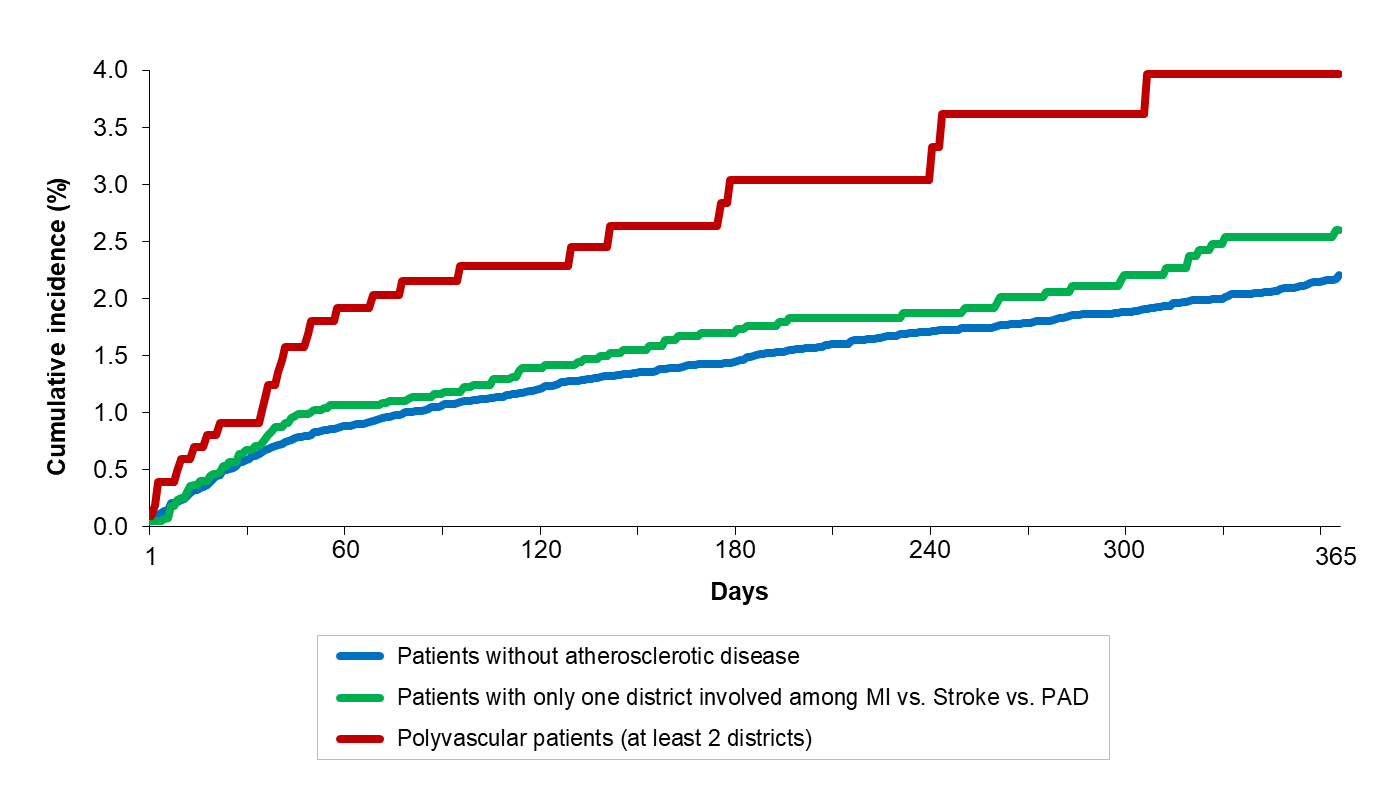
**

**Supplementary Figure 1. Kaplan-Meier estimates for venous thromboembolism (VTE) recurrences over one year:** This graph depicts the cumulative incidence of VTE recurrence in patients without atherosclerotic disease (blue line), with single vascular artery disease (green line), and with polyvascular disease (red line). A log-rank test revealed statistically significant differences between the groups (p-value = 0.001).

**Supplementary Figure 2. Kaplan Meier curves for all-cause death**

**
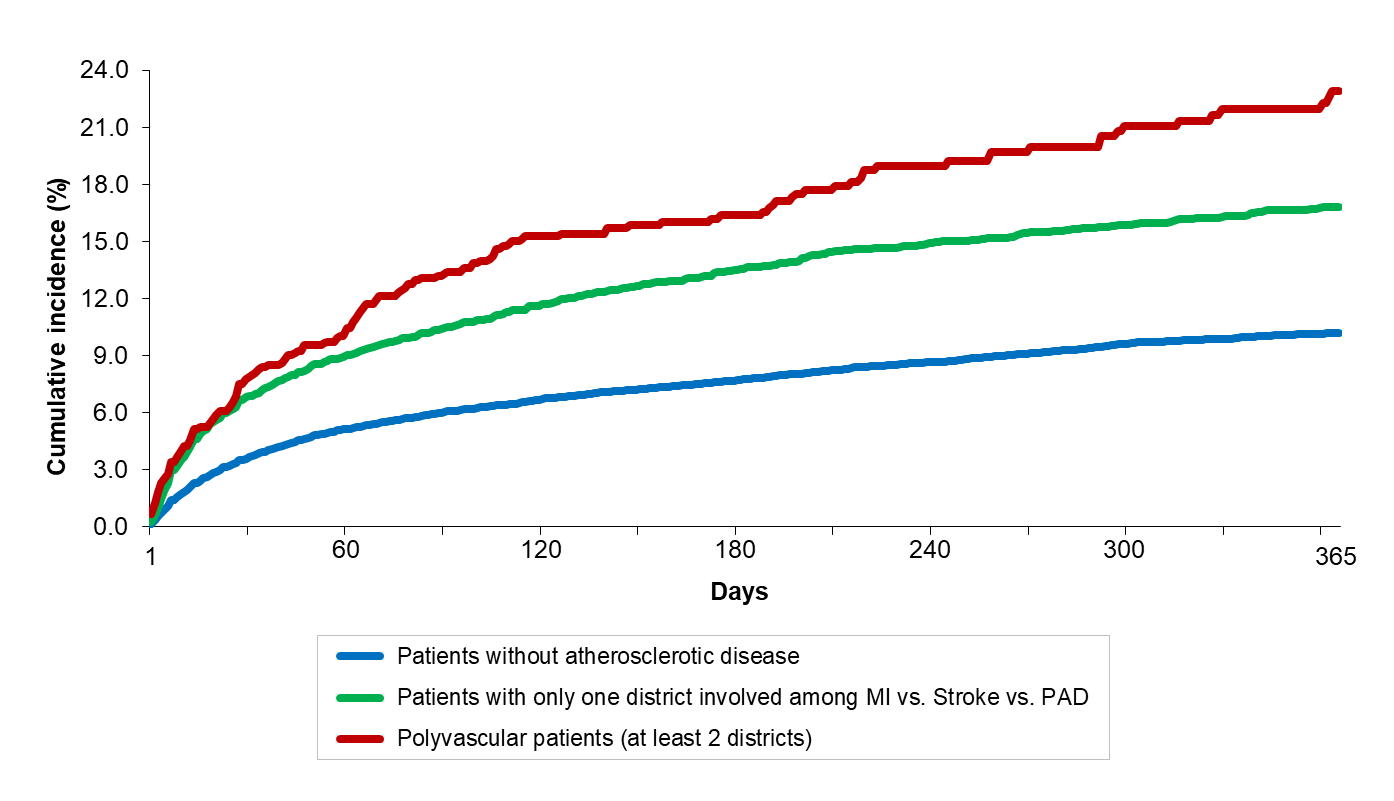
**

**Supplementary Figure 2. Kaplan-Meier estimates for all-cause death over one year: This graph depicts the cumulative incidence of all-cause death in patients without atherosclerotic disease (blue line), with single vascular artery disease (green line), and with polyvascular disease (red line). A log-rank test revealed statistically significant differences between the groups (p-value < 0.001)**

**Supplementary Figure 3. Kaplan Meier curves for major bleeding**

**
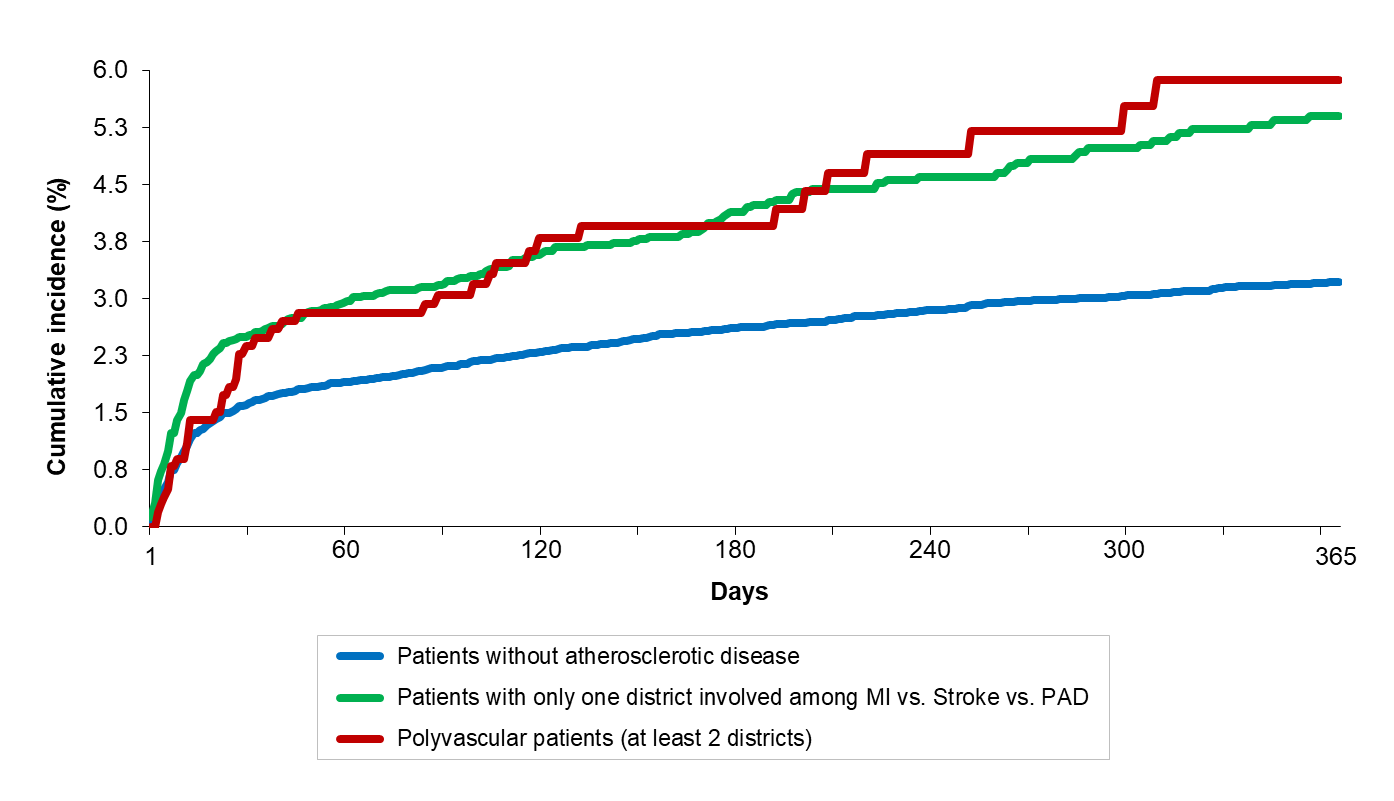
**

**Supplementary Figure 3. Kaplan-Meier estimates for major bleeding over one year:** This graph depicts the cumulative incidence of major bleeding in patients without atherosclerotic disease (blue line), with single vascular artery disease (green line), and with polyvascular disease (red line). A log-rank test revealed statistically significant differences between the groups (p-value < 0.001).
